# Jawbone remodeling: a conceptual study based on Synchrotron High-resolution Tomography

**DOI:** 10.1038/s41598-020-60718-8

**Published:** 2020-03-02

**Authors:** Giovanna Iezzi, Carlo Mangano, Antonio Barone, Federico Tirone, Luigi Baggi, Giuliana Tromba, Adriano Piattelli, Alessandra Giuliani

**Affiliations:** 1 Department of Medical, Oral and Biotechnological Sciences, University of Chieti-Pescara, Chieti Scalo, CH Italy; 2 Private Practice, Gravedona (CO), Italy; 3 Department of Medical, Surgical, Molecular and of the Critical Area Pathologies, University of Pisa, Pisa, Italy; 4 Private Practice, Cuneo, Italy; 5 Department of Social Dentistry, National Institute for Health, Migration and Poverty, Rome, Italy; 6 School of Dentistry, University of Rome “Tor Vergata”, Rome, Italy; 7 Elettra Sincrotrone Trieste S.C.p.A, Trieste, Italy; 8 Chair of Biomaterials Engineering, Catholic University of Murcia (UCAM), Murcia, Spain; 9 Villa Serena Foundation for Research, Città Sant’Angelo (Pescara), Italy; 10 Department of Clinical Sciences, Polytechnic University of Marche, Ancona, Italy

**Keywords:** Restorative dentistry, Biomedical engineering

## Abstract

One of the most important aspects of bone remodeling is the constant turnover mainly driven by the mechanical loading stimulus. The remodeling process produces changes not only in the bone microarchitecture but also in the density distribution of the mineralized matrix - i.e. in calcium concentrations- and in the osteocyte lacunar network. Synchrotron radiation-based X-ray microtomography (microCT) has proven to be an efficient technique, capable to achieve the analysis of 3D bone architecture and of local mineralization at different hierarchical length scales, including the imaging of the lacuno-canalicular network. In the present study, we used microCT within a conceptual study of jawbone remodeling, demonstratively focusing the investigation in two critical contexts, namely in the peri-dental and the peri-implant tissues. The microCT analysis showed that a relevant inhomogeneity was clearly present in both peri-dental and peri-implant biopsies, not only in terms of microarchitecture and mineralization degree, but also considering the lacunar network, i.e. size and numerical density of the osteocyte lacunae. The correlated histological results obtained on the same samples confirmed these observations, also adding information related to non-mineralized tissues. Despite its demonstrative nature, it was concluded that the proposed method was powerful in studying jawbone remodeling because it revealed a direct correlation of its rate with the lacunar density, as achieved by the analysis of the osteocyte lacunar network, and an inverse correlation with the local bone mineral density, as revealed with the Roschger approach.

## Introduction

Remodeling is a process enabling bone tissue to adapt to different physiological conditions and to replace damaged bone with newly-formed bone^1^, producing complete renewal of the entire skeleton mass about every 10 years^2^. Several studies have been focused on regenerative properties of bone, but few of them were referred to the jaw sites. Conversely, it must be considered that the jawbone presents unique properties: thus, data regarding other skeletal bones, many of them referred to long bones^3–7^, may not be completely applicable to the jaw^8^.

Indeed, it is known since long time that the jawbone is remodeled faster than the other skeletal bones^9^. This seems to be due to jaw morphogenesis: jaw arises from neural crest cells of the neuroectoderm germ layer and not of the mesoderm;^10^ moreover, it undergoes intramembranous, instead of endochondral, ossification^11^. Regarding the stem cells derived from the jaw, bone marrow stromal cells exhibit higher osteogenic potential and different characteristics respect to stem cells recruited in other skeletal bones^12–16^.

Consequently, because of the aforementioned features of the jawbone matrix, even if several studies have been focused on bone remodeling in other skeleton sites, there is a special need of specific investigations referred to the jawbone.

One of the most important aspects of jawbone remodeling is the constant turnover mainly driven by the mechanical loading stimulus (forces) during mastication; this remodeling is particularly evident in the alveolar bone^1,17–19^. Furthermore, jawbone remodeling has been demonstrated to be a mechanism by which bone is able to prevent the accumulation of microdamages, with an extension of the bone fatigue parameters, determining an augmented biomechanical competence^18^. In this context, osteocyte activity was recently shown to be regulated not only by biological but also by mechanical signals driven by the above-mentioned loading forces^20^. Osteocytes reside in cavities called lacunae that are interconnected through cellular dendrites that extend into small channels called canaliculi. For many years it has been hypothesized that the morphology of the lacuno-canalicular network (LCN) is related to the processes of mechanotransduction of osteocytes^21^. In turn, several evidences that the osteocyte network organization could control or influence modeling and remodeling were found^22^. If these evidences will be definitely proved, the influence of the osteocyte network organization on bone remodeling processes would be crucial, determining three important consequences: the calcium regulation, the microdamage repair, and a mechanically adaptive control of bone architecture.

Moreover, the LCN was shown to be the main actor in bone mechanical answer to loading^17,18,23–27^. Indeed, it was recently shown that a significantly higher number of osteocytes was present around immediately loaded human dental implants with respect to control sites left to heal submerged (i.e. without their contribute to masticatory loading)^17^, indicating possible correlations between masticatory loading and number of osteocytes, and a key role of these cells in remodeling of peri-implant bone^17^. It was also shown^28^ that a high quantity of compact, lamellar bone was found in peri-implant bone of loaded implants. These previous investigations were performed by means of histology; however, as it was recently shown^29,30^, the gold-standard for studying bone remodeling in the jaw districts is, probably, a combination of two-dimensional (2D) histologic and three-dimensional (3D) methods of imaging.

In this framework, synchrotron-based X-ray microtomography (microCT) has proved to be a powerful technique^31^ not only to perform non-destructive quantification of bone mineral density, obtaining 3D bone architecture and local mineralization analysis at different hierarchical scales of length^32–34^, but also to access sub-micrometric resolutions with imaging of the lacuno-canalicular networks^35^.

More specifically, two literature studies performed with microCT were considered as a premise to this conceptual study. In the first, Roschger *et al*.^36^ described an appropriate method to sensitively measure the bone mineralization density distribution (BMDD) in bone biopsies, demonstrating its efficiency as a support to diagnosis and treatment of several bone diseases. In the second study, Hesse *et al*.^8^ found that the apparent BMDD of jawbone is significantly smaller than that of tibia, coherently with the higher bone turnover of jawbone^9^. Moreover, they also showed that the variance of the lacunar volume distribution was significantly different depending on the anatomical site^8^.

In this context, very recently, lacunar morphology and distribution in human jawbone, obtained from patients affected by medically-related osteonecrosis (MRONJ), was evaluated^8,37,38^ in pioneering studies showing that synchrotron radiation microCT could reliably map the changes of the mineralized matrix for a better understanding of MRONJ pathogenesis.

Thus, the aim of the present demonstrative study was to propose an innovative method of analysis, on a multiple length-scale, of the human jaw, aimed at the study of bone remodeling and based on synchrotron radiation high-resolution tomography (microCT). The information obtained by microCT was cross-linked with histological data on the same samples to confirm microCT observations, also adding data related to non-mineralized tissues. This original approach was based on the rationale that, since the osteocytes are actively involved in the remodeling processes^17,18^ and in the maintenance of the bone matrix^20^, it should be possible to study remodeling through high-resolution imaging and a combined quantitative analysis of the BMDD and of the osteocyte lacunar network. In particular, osteocytes are cells that form from osteoblasts after their inclusion into the bone matrix; therefore, we hypothesized that the morphology and the distribution of the osteocyte lacunae were modified in the peri-dental and peri-implant bone. We selected these jaw sites in order to validate the proposed method of analysis that was shown to provide, for the first time to the authors’ knowledge, unique insights on questions on how the jaw remodeling and its rate are related both to the microarchitecture of the lacunar network of osteocytes and to the local bone mineral density.

## Results

**Figure 1:**
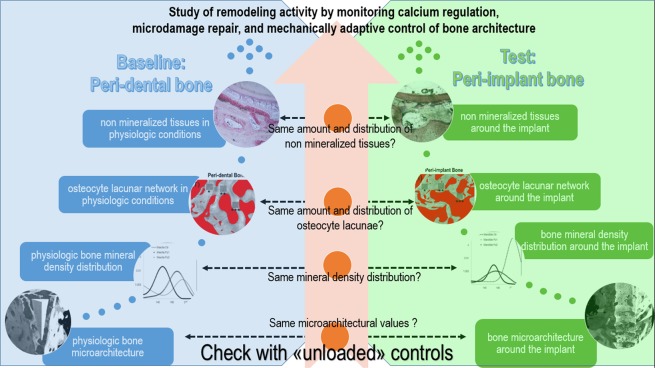
Flow chart of the overall study design. The study responds, with the rigorous method described here, to the scientific need to correlate osteocytes and remodeling activity, with particular reference to calcium regulation, microdamage repair, and mechanically adaptive control of bone architecture^22^.

Literature agrees that a jawbone site, after a tooth extraction and at least 6 months of time, is perfectly healed in a patient not suffering from other diseases^39,40^. However, after extraction of the tooth and during the healing time, these sites do not participate in mastication, possibly generating differences with the physiological conditions of the peri-dental bone and/or with the testing case of the peri-implant bone. This divergence could be expressed in terms of calcium amount and distribution and/or bone architecture at different length-scales, including at the level of the osteocyte lacunar network, justifying the relevance of our study design, graphically summarized in the flow chart in Fig. 1.

**Figure 2:**
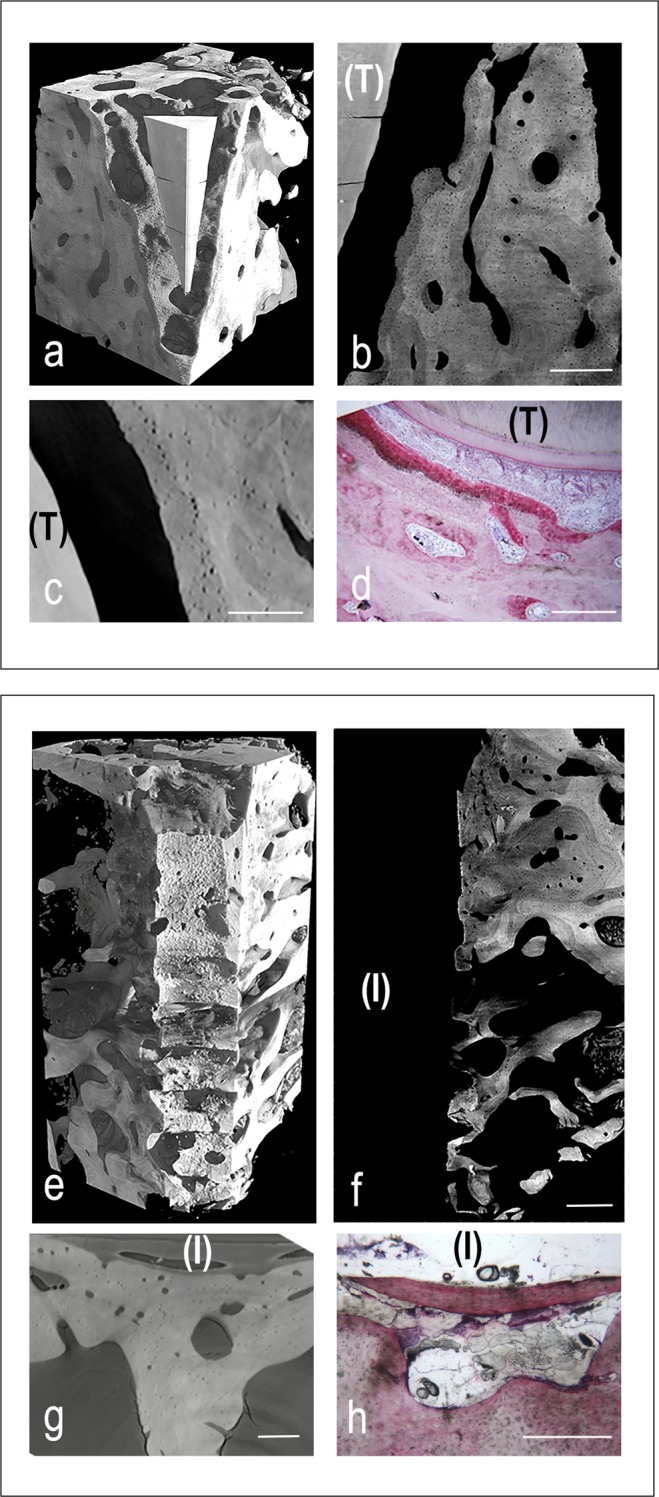
Cross-linking of microCT and histological images in peri-implant (panels a-d) and peri-dental (panels e-h) representative sites of the retrieved samples. (**a–d**) Peri-implant bone site: (**a**) MicroCT 3D reconstruction. The titanium implant was previously removed (with a method preventing the bone/implant interface damaging) to achieve the correct X-ray transmission through the sample during the SR-microCT acquisition; (**b**) MicroCT 2D longitudinal section. Inhomogeneity in mineralization degree and bone ultrastructure throughout the sample was clearly shown. Bar: 300 µm; (**c**) MicroCT 2D transversal section. Bar: 180 µm; (**d**) Histologic 2D transversal section. Original magnification: 40×. Bar: 100 µm (**e–h**) Peri-dental bone site: (**e**) MicroCT 3D reconstruction; (**f**) MicroCT 2D longitudinal section: tooth residual with peri-dental bone behind it. Like in peri-implant sites, evidences of Inhomogeneity in mineralization degree and bone ultrastructure were shown. Bar: 300 µm; (**g**) MicroCT 2D transversal section. Bar: 250 µm; (**h**) Histologic 2D transversal section. Original magnification: 100×. Bar: 50 µm (**T**): tooth residual; (**I**) Implant site, after the implant removing.

MicroCT and histological images of representative peri-dental and peri-implant bone samples were shown in Fig. 2. All tissues, but mineralized bone, have been made virtually transparent. The 3D reconstructions were shown in Fig. 2a,e, for peri-dental and peri-implant biopsies, respectively. Representative longitudinal and transversal sections were respectively reported in Fig. 2b,c, for peri-dental sites, and in Fig. 2f,g, for peri-implant ones.

**Figure 3:**
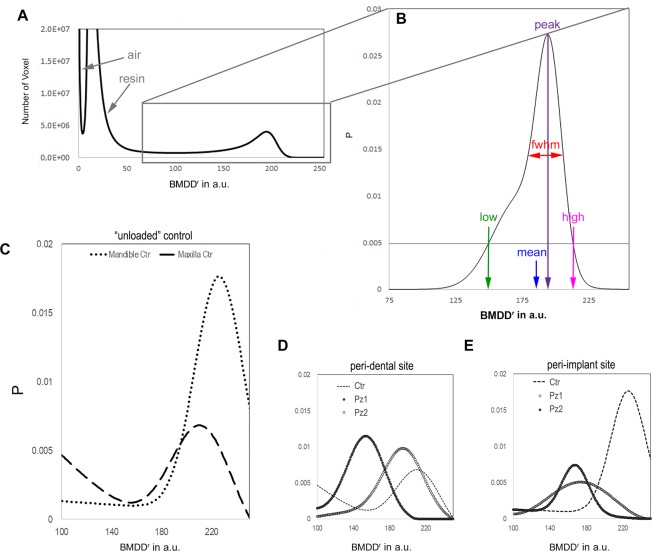
Relative bone mineral density distribution (BMDD^r^) of the mineralized bone. (**A**) The peaks indicated by the arrows are referred to the air outside the sample and to the resin used to include the biopsy. (**B**) The parameters derived from the Roschger approach^36^ are indicated. The threshold of P = 0.005 was chosen because it is a good compromise between maintaining good sensitivity for low and high values in the BMDD^r^ and minimizing potential artifacts due to partial volume effect in the evaluation of BMDD^r^_low_. (**C**) BMDD^r^ are shown for the control unloaded samples. The slightly reduced mean value, BMDD^r^_mean_, in the maxillary region with respect to the mandible site suggests a region-dependent behavior of the BMDD^r^. (**D**) BMDD^r^ profiles are shown for the peri-dental samples, namely the Pz1 and the Pz2 biopsies, when compared with the unloaded Ctr. Inhomogeneous distributions were detected in the two peri-dental biopsies; however, in both the samples the mineralization values, i.e. the Ca concentration, were found to be reduced with respect to the unloaded Ctr. (**E**) BMDD^r^ profiles are shown for the peri-implant tests, namely the Pz1 and the Pz2 biopsies, when compared with the unloaded Ctr. Similarly to the peri-dental sites, a strongly inhomogeneous density distribution was detected in the two peri-implant biopsies, in both the samples reduced with respect to the unloaded Ctr.

The first level of study was focused on peri-dental and peri-implant bone microarchitecture and on the relative bone mineral density distribution (BMDD^r^). Several subvolumes, collected in different areas and fully included in the biopsies, were selected, producing the microarchitecture data reported in Table 1. The same subvolumes were also investigated for BMDD^r^ mapping: the complete set of indices, derived from the profile fitting, was reported in Table 1. Rationale and results referred to BMDD^r^ were shown in Fig. 3.

**Table 1:** 1st level of analysis: study of the peri-implant/peri-dental bone microarchitecture and of the relative bone mineral density distribution (BMDD^r^) of the mineralized bone.

	peri-dental bone			peri-implant bone		
	Pz1	Pz2	Ctr	Pz1	Pz2	Ctr
BS/BV [mm^−1^]	12.0 (2.8)	12.0 (1.5)	21.0 (0.1)	19.5 (9.2)	20.5 (0.7)	14.5 (2.1)
BV/TV [%]	57.8 (0.9)	57.7 (0.4)	42.2 (5.4)	40.1 (16.5)	31.8 (2.5)	54.2 (6.6)
Tb.Th [µm]	165 (38)	168 (43)	104 (16)	119 (59)	97 (1)	136 (18)
Tb.Nr [mm^−1^]	3.5 (0.7)	3.2 (0.6)	4.0 (0.0)	3.5 (0.7)	3.0 (0.0)	4.0 (1.4)
Tb.Sp [µm]	118 (23)	123 (21)	141 (10)	170 (31)	207 (22)	119 (47)
Tb.DA	0.440 (0.022)	0.606 (0.075)	0.752 (0.010)	0.520 (0.074)	0.515 (0.168)	0.482 (0.061)
Tb.Conn.D [mm^−3^]	82 (13)	30 (6)	60 (56)	78 (34)	64 (8)	89 (5)
BMDD^r^_mean_	154	195	212	175	168	226
BMDD^r^_peak_	153	193	216	164	168	222
BMDD^r^_fwhm_	50	52	59	111	35	46
BMDD^r^_low_	104	143	153	64	133	180
BMDD^r^_high_	>255	203	>255	204	247	>255

In the three-dimensional morphometric analysis of the retrieved biopsies, mean and standard deviation of the investigated parameters are listed. In the study of the BMDD^r^, the parameters deriving from the profile fitting are indicated. Ctr: bone biopsies retrieved after spontaneous healing (i.e. at least 12 months after tooth-extractive procedure) and before implant loading.

**Figure 4:**
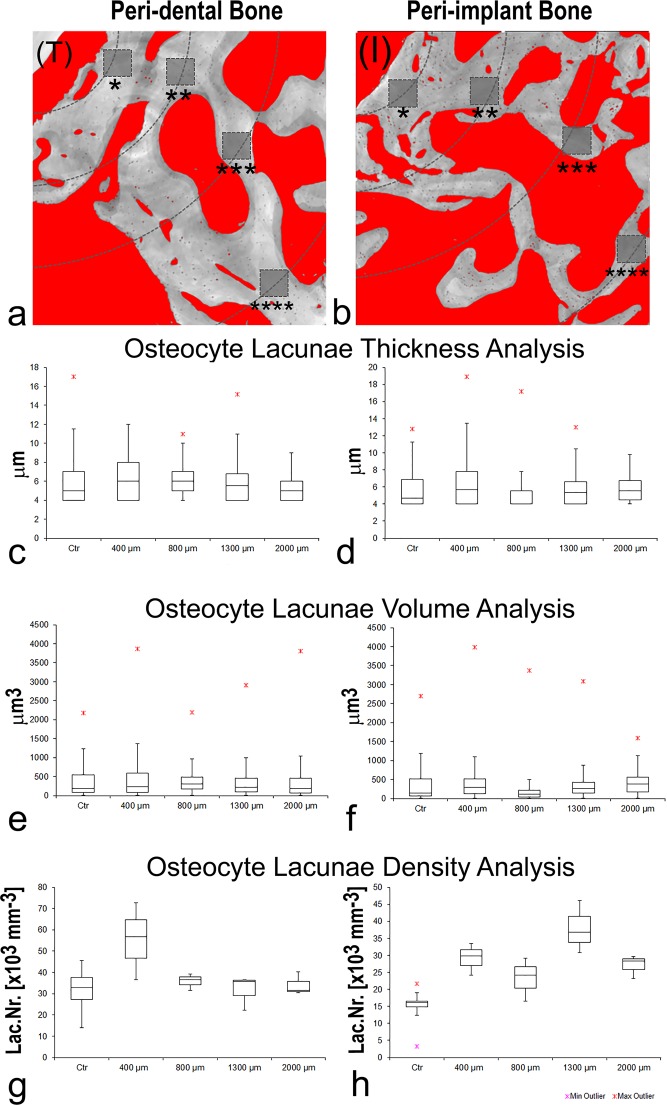
2nd level of study: osteocyte lacunar network. (**a-b**) Sampling 2D microCT slices: (**a**) peri-dental bone; (**b**) peri-implant bone. The histograms have been segmented according to the application of the Mixture Modeling algorithm. Red phase: organic tissues; graded grey phase: mineralized bone. The dotted circumference arcs indicate the different distances of the investigated sites from the tooth/implant surface: namely at 400 µm (*), 800 µm (**), 1300 µm (***) and 2000 µm (****). (T): tooth residual; (I) Implant site, after the implant removing. (c-f) Box-plots graphically depicting groups of investigated volumes at the different distances from the tooth/implant surface through their quartiles: (c-d) osteocyte lacunae thickness (Lac.Th) analysis in peri-dental (**c**) and peri-implant (**d**) sites; (**e-f**) osteocyte lacunae volume (Lac.V) analysis in peri-dental (**e**) and peri-implant (**f**) sites; (**g-h**) osteocyte lacunae density (Lac.Nr) analysis in peri-dental (**g**) and peri-implant (**h**) sites.

A second and more sophisticated level of study was also achieved, performing the 3D morphometric analysis of the osteocyte lacunae in all the biopsies, as shown in Table 2. Solely for this analysis, data obtained from the two patients involved in peri-dental bone study and from the two patients involved in peri-implant bone study were unified and renamed peri-dental@Test and peri-implant@Test, respectively. Interestingly, standard deviations were quite high, suggesting a repetition of the same investigation, but with a correlation of data to the distance from tooth or titanium implant surface, as shown in Fig. 4. Sampling slices in the peri-dental and in the peri-implant bones were shown in Fig. 4a and in Fig. 4b, respectively. Several subvolumes, approximately 8 × 10^6^ µm^3^ each, were selected at different distances from tooth/implant surface, namely at 400 µm, 800 µm, 1300 µm and 2000 µm, as shown with the dotted circumference arcs in Fig. 4a,b. Box-plots were used to graphically depict groups of investigated volumes through their quartiles. The distribution of the osteocyte lacunar thickness, volume and density was evaluated in peri-dental (Fig. 4c,e,g) and peri-implant (Fig. 4d,f,h) sites, respectively.

**Table 2:** 2nd level of study: three-dimensional morphometric investigation of the osteocyte lacunae in the retrieved jaw biopsies.

	peri-dental bone		peri-implant bone	
	*Test*	*Ctr*	*Test*	*Ctr*
*Lac.Th [µm]*	5.2 (1.3)	5.6 (0.7)	5.7 (0.6)	5.6 (0.5)
*Lac.V [µm*^3^]	409 (180)	371 (133)	410 (125)	420 (197)
*Lac.Nr* [*×*10^3^ *mm*^*−3*^]	31.4 (10.6)	25.6 (6.9)	23.5 (6.0)	11.3 (5.0)

*Ctr*: bone biopsies from the same region of the *Test*, retrieved after spontaneous healing (i.e. at least 12 months after tooth-extractive procedure) and before implant loading. Mean values (± standard deviation) of the investigated parameters are listed.

### Peri-dental bone

The study of the peri-dental bone should be the baseline for the evaluation of the proposed demonstrative protocol. Indeed, the studied peri-dental biopsies represent the physiological condition of the jawbone, containing the whole periodontium that surrounds and supports the teeth, maintaining them in the jawbones.

By comparison between peri-dental bones (Pz1 and Pz2) and their unloaded controls, both of them located in maxilla, it was possible to observe that BV/TV and TbTh increased in the peri-dental bones with respect to controls of around 37% and 60%, respectively; in the meantime, BS/BV, TbSp and DA were reduced of around 43%, 15% and 31%, respectively.

BMDD^r^ profiles of peri-dental samples (Pz1 and Pz2) and of maxillary unloaded control were shown in Fig. 3D. Moreover, as reported in Table 1, although similar data were found in terms of density variance (BMDD^r^_fwhm_), smaller BMDD^r^_mean_ and BMDD^r^_peak_ values were obtained in both peri-dental biopsies with respect to unloaded controls, with reduction of 18% and 20%, respectively.

As reported in Table 2, the 3D morphometric analysis of the osteocyte lacunae revealed similar values in Test and in Control sites, when evaluating the mean Lac.V, Lac.Th and Lac.Nr. In agreement with the previous results, box-plots referred to osteocyte lacunar thickness and volume revealed comparable median values at the different distances and with respect to unloaded samples, as shown in Fig. 4c,e, respectively. Conversely, box-plots related to lacunar density were the most interesting: comparable values to unloaded control samples were found at all the different distances with the exception of the nearest (400 µm) distance to the tooth, where the lacunar density was sensibly higher, i.e. with a number of lacunae that was almost twice compared to control and other distances. This result is fully confirmed by observing the interface tooth/bone in the stack-sequence of 2D axial slices (subvolume: 560 × 2700 × 950 μm^3^) that has been reported as representative for Pz1 in Movie 1 of the Supplementary Material section.

The histologic data showed, at lower magnification, the root surrounded by alveolar bone (Fig. 2h). The periodontal ligament, rich of small blood vessels, was interposed between cementum and compact bone, which is the portion of bone that lines the tooth socket. It appeared intensely stained with acid fuchsin, featuring the presence of newly formed bone. In this area, many osteocytes with large osteocyte lacunae were present, confirming the microCT observations of the osteocyte network and the related quantitative data. The remaining alveolar bone, more distant from the tooth, consisted of mature bone, remodeling areas and small marrow spaces lined by osteoblasts, deposing osteoid matrix that was detected in close proximity to small blood vessels. Histomorphometry showed that the percentage of newly formed bone, mature bone and bone morrow was respectively around 22%, 31% and 47%, as shown in Table 3.

**Table 3:** Histomorphometric data: percentages of newly-formed bone, mature bone and morrow spaces in the different groups of study.

	peri-dental bone		peri-implant bone	
	*Test*	*Ctr*	*Test*	*Ctr*
*New bone [%]*	21.5 (2.1)	10.9 (2.2)	18.8 (0.7)	12.2 (0.9)
*Mature bone [%]*	30.9 (2.3)	31.0 (1.0)	41.2 (2.1)	41.3 (1.3)
*Bone morrow [%]*	47.5 (3.4)	56.8 (2.2)	40.0 (2.5)	46.7 (1.7)

Mean values (± standard deviation) are indicated.

### Peri-implant bone

The study of the peri-implant bone was considered the fundamental test in the proposed demonstrative protocol: the presence of the titanium implant, the absence of the tooth and of the periodontium were expected to generate divergences in the jawbones with respect to the respective unloaded controls but also with respect to the previous case of the peri-dental bone.

When comparing peri-implant bone (Pz1 and Pz2) and the respective unloaded controls, it was possible to observe that BV/TV and TbTh slightly decreased in the former, with reductions of around 33% and 20%, respectively; in the meantime, BS/BV and TbSp increased in the former with respect to unloaded controls and with percentages of around 33% and 58%, respectively. However, the standard deviations of the test (peri-implant) samples were so large as to make challenging any comparative evaluation with the unloaded control.

BMDD^r^ profiles of peri-implant samples and of the unloaded controls are shown in Fig. 3E. Similarly to the case previously described (peri-dental bone), smaller BMDD^r^_mean_ and BMDD^r^_peak_ values were obtained in the two peri-implant biopsies when compared to the unloaded controls, with reduction of 24% and 25%, respectively. These data indicate that the unloaded controls have, in different areas of the biopsies, higher calcium percentages than the peri-implant sites. However, differently from peri-dental sites, non-homogeneous data were found in peri-implant biopsies for BMDD^r^_fwhm_, with a wider density distribution in Pz1 when compared to Pz2.

The 3D morphometric analysis of the osteocyte lacunae revealed similar values in Test and in Ctr sites, when evaluating Lac.V and Lac.Th; however, relevant mismatches were obtained in the evaluation of Lac.Nr with higher values in the Test than in the Ctr, as reported in Table 2. Similarly to the peri-dental samples, box-plots referred to osteocyte lacunar thickness revealed comparable median values at the different distances and with respect to unloaded samples. However, the 75th percentile (Q3) values were higher at the nearest distance (400 µm) from the titanium implant surface in comparison both to the other distances and to the control unloaded samples. Box-plots related to lacunar density showed interesting results also in peri-implant districts: indeed, high variability at the different distances was found, even if median values were comparable to maxillary ones at distances more far than 400 µm. Moreover, it was confirmed here that the absence of loading in control-samples decreased the numerical density of the osteocyte lacunae (Lac.Nr), with a number of lacunae that was almost half compared to the peri-implant districts. All these data can be observed in the stack-sequence of 2D axial slices (subvolume: 2500 × 1600 × 1000 μm^3^) that has been reported as representative for Pz1 in Movie 2 of the Supplementary Material section.

The histologic analysis showed, at lower magnification, that bone tissue was present around the implant surface in coronal and middle portion of the implant (Fig. 2d). Specifically, the presence of immature osteoid matrix, woven bone and lamellar bone in areas close to the implant surface was detected, proving that active bone formation and bone remodeling were still in progress. At higher magnification, in agreement with the microCT data, in bone close (~500 µm) to the implant, many osteocytes within large lacunae and small marrow spaces colonized by blood vessels were present. Around and inside the implant threads, osteons were detected and, in some cases, they were in tight contact with the implant surface. Histomorphometry showed that the percentage of newly formed bone, mature bone and bone morrow was respectively around 19%, 41% and 40%, as shown in Table 3.

## Discussion

Recent advances in synchrotron-based microCT allowed to achieve unprecedented 3D imaging of the osteocyte lacunar network in different sites of interest^41^. Osteocyte lacunar density and morphology were studied in different loading conditions, according to the mechanical axis of the bone^42^, at different ages^43,44^ and in different skeletal sites (femur, tibia and iliac crest). However, few studies were focused on microCT investigation of osteocyte lacunar morphology and density in jaws, and all of them were referred to MRONJ patients^8,37^.

In this context, the present demonstrative study was designed to find, for the first time to the authors’ best knowledge, possible correlations between bone multi-length scale architecture and its remodeling in peri-dental and peri-implant jaw sites. This was achieved by means of synchrotron-based microCT and with the support of histology, combining the analysis of mineralized bone density distribution, bone microarchitecture and lacunar network.

This analysis was performed through two levels of study: the first level was focused on bone microarchitecture and on the study of the mineralized bone density distribution, with the support of the innovative Roschger approach^36^; the second level was focused on the lacunar network, analyzing the osteocyte lacunae size and distribution.

In peri-dental sites, an increased specific volume and trabecular thickness, together with reduced specific surface, trabecular spacing and anisotropy degree, with respect to the unloaded control were found. These results coherently indicate that the “unloaded controls”, already after few months in absence of masticatory loading, present an unbalanced microarchitecture with a reduced bone mass and trabeculae smaller and more oriented, with trends mimicking osteoporotic conditions. Possibly, differently from peri-dental sites, the absence of the periodontal complex (i.e. of the periodontal ligament and of the cementum) in these control samples has further increased this trend. Most likely, the periodontal ligament tissues combined to bone loading guaranteed in peri-dental biopsies the maintenance of surrounding bone microarchitecture, while the unloading conditioning in control-samples seemed to have affected its architecture because of the unbalanced bone formation and resorption, also confirmed by histomorphometrical analysis, which showed higher values of the mineralized components compared to bone marrow.

From the comparison between peri-implant bone and the respective unloaded control, reduced specific volume and trabecular thickness and increased specific surface were found in the former when compared to the unloaded control. These results must be considered with caution, due to the high standard deviations found in test sites (Pz1 and Pz2) that are motivated by biopsies heterogeneity in terms of volume, with areas sometimes also far from the implant site and therefore with a lower number of remodeling areas.

Bone remodeling also results in a heterogeneous distribution of mineralized tissue units, with variable degrees of mineralization. This heterogeneity can be assessed through the study of bone mineralization density distribution by using advanced techniques such as synchrotron radiation microCT^35–38,45^; moreover, the degree of mineralization is a quality factor that influences the mechanical properties of bone^46^. Our results referred to the study of bone mineralization density distribution by microCT are consistent with and extend the results of Hesse *et al*.^8^: they found that mean, peak, low, and high values of the BMDD^r^ of jawbone were significantly smaller and fwhm values significantly larger compared to the respective parameters of tibia, showing a higher bone remodeling in the jawbone than in tibia. With a similar method we found smaller mean and peak values, which correspond to reduced calcium concentrations and distributions, in both peri-dental and peri-implant biopsies when compared to unloaded controls, demonstrating that the masticatory loading in the former cases induces an increased remodeling. Again, these results were confirmed by histomorphometric analysis, as the percentage of newly formed bone was higher in peri-dental and peri-implant bone than in unloaded controls. The non-homogeneity found in peri-implant biopsies, in terms of mineralized bone density variance, can be motivated in the same way of the increased standard deviation in the microarchitecture study. In particular, in peri-implant biopsies, a wider density distribution was found in Pz1 when compared to Pz2. This can be explained considering the reduced transversal dimensions of the Pz2-biopsy, with fewer areas at a great distance from the implant and therefore with less areas with high calcium content (mature bone) than the Pz1-biopsy.

Undoubtedly, the most interesting results were found through the study of second level, referred to the analysis of the osteocyte lacunae size and distribution. Hesse *et al*.^8^ showed by microCT that the variance of the lacunar volume distribution was significantly different depending on the anatomical site (tibia, femur or jaw). Moreover, by the comparison between MRONJ and control jawbone, they found a significant decrease in osteocyte-lacunar density in the former group, with no evident variation of osteocyte-lacunar volume distribution between the two groups of study. Interestingly, our analysis of the lacunar mean data seemed to indicate that the unloaded condition resembles the lacunar architecture of the jawbones affected by MRONJ; indeed, even if the unloaded condition was shown not affect the mean volume of the osteocyte lacunae, an higher lacunar density was obtained in peri-implant biopsies than in the respective unloaded controls. These data may mean that, although the absence of loading in Ctr-samples did not affect the size of the osteocyte lacunae, it negatively influenced the number of these, suggesting the use of immediately-loaded implants. Indeed, our results are also in agreement with another histological study in which, with statistical significance, higher osteocyte density was found around immediately-loaded implants compared to unloaded bone sites. The greater number of osteocytes in the bone around the implants immediately-loaded could be related to the functional adaptation required by the upload stimulus, which also explains the hypothesized involvement of osteocytes in maintaining the bone matrix^17^. In fact, the network of osteocytes is the cellular structure that allows bones to meet local needs in terms of bone formation and bone resorption (ie the bone remodeling process), responding to mechanical needs. In turns, the mechanical loading affects bone remodeling, whereby osteocytes are thought to function as mechanosensors in bone^20^.

The analysis of the osteocyte lacunar density versus the distance from the tooth/implant confirmed that peri-implant bone had higher lacunar density than its unloaded control. More interestingly and for the first time to the authors’ knowledge, this analysis also showed that, in peri-dental bone, at the nearest (400 µm) distance from the tooth, the density of lacunae was sensibly higher compared to longer distances and compared to the unloaded control, most likely because of the presence of periodontal tissues at the interface between the tooth and the bone. Indeed, the alveolar bone is the bone that surrounds the roots of the teeth, forming bone sockets; specifically, the socket walls are covered by bundle bone (200–400 µm in thickness) that is a periodontium component^47^. Moreover, a higher lacunar density near the tooth compared to the other distances is also justified the greater proximity of this site to the loading area, i.e. the tooth, confirming the fundamental role of loading in bone remodeling.

In conclusion, in both the peri-dental and the peri-implant sites, it was observed that the presence of load with respect to the unloaded control samples increased the numerical density of osteocyte lacunae and decreased the number of areas with high percentages of calcium (mature bone). The histologic data confirmed this conclusion: indeed, no osteoblasts, osteoid matrix or osteoclasts activity was found in control samples, most likely because unloaded bone was submitted to reduced remodeling than loaded sites, i.e. peri-dental and peri-implant bone.

The inherent limitation of this conceptual study is the small sample size; thus, additional data are needed in the future to confirm the previous observations. Indeed, the scarce availability of biopsies with identical a priori conditions forced us to select the peri-dental bone from sites of maxillary origin and the peri-implant bone from mandible, with only two elements per group. However, the reduced number of samples and the different anatomical positions (maxilla and mandible) did not affect the demonstrative character of the study; the possibility of studying “unloaded controls” coming from the same regions (maxilla and mandible) allowed this demonstrative study to be carried out successfully.

Moreover, the comparison between (unloaded) maxilla and mandible controls, through the proposed protocol and with the support of data found in literature, allowed a further confirmation of the reliability of the proposed method; indeed, reduced specific volume (BV/TV) and mean thickness (TbTh) were found in the maxilla, as observed in previous studies on dogs^46^. Furthermore, as shown in Fig. 3C, slightly reduced BMDD^r^_mean_ and BMDD^r^_peak_ values in maxillary region with respect to mandible sites confirmed a region-dependent behavior of the mineral density distribution, i.e. of the calcium concentration and distribution (Ca weight %), as already observed in different skeletal sites^36^. Interestingly, the maxillary unloaded control also presented higher lacunar density than the mandibular unloaded control, confirming that these sites may adapt to components of physical forces by altering remodeling rates.

In conclusion, synchrotron-based phase-contrast microCT was shown to be a powerful method in studying jawbone remodeling, revealing, for the first time to the authors’ knowledge, a direct correlation of its rate with the lacunar density, as obtained by the analysis of the osteocyte lacunar network, and an inverse correlation with the local bone mineral density, as revealed by the Roschger approach. Moreover, an increased jawbone remodeling was also associated to masticatory loading, suggesting the use of immediately loaded dental implants in surgery.

## Methods

### Sample collection and permissions

Eight patients were included in this study: two of them provided peri-dental biopsies, represented by teeth extracted with a small portion of alveolar bone (Maxilla-Pz1 and Maxilla-Pz2) and located in maxillary sites; other two patients provided peri-implant bone biopsies (Mandible-Pz1 and Mandible-Pz2) that were located in mandible and were studied after gently removing of the titanium implant; the remaining four patients provided “unloaded” control bone biopsies, two from edentulous maxillary (Maxilla-Ctr) and two from edentulous mandibular (Mandible-Ctr) sites. Peri-dental, peri-implant and the respective “unloaded” biopsies were selected in premolar regions. The “unloaded” control bone biopsies were retrieved at least 12 months after tooth-extractive procedure (during the implant surgery), after spontaneous healing. All these control sites, being edentulous and consequently not participating in the mastication for 1 year before implant rehabilitation procedure, were considered as “unloaded”, since other loads, like bone weight, are negligible compared to the masticatory one^48^.

All patients gave written informed consent and the permissions of two Human Investigation Committees were granted (Ethics Committee of Hospital of Varese, Prot. 826–03/10/2013; Ethics Committee of A.S.O. Santa Croce e Carle-Cuneo, Prot. 07/14–17/12/2014). The experiments were performed in full agreement with relevant guidelines and regulations.

The choice of different anatomical sites (maxilla and mandible) and the restricted number of elements (nr=2 per group of study) was limited by the difficulty in finding such patient samples, fully respecting inclusion criteria imposed by the two Human Investigation Committees.

Retrieved bone cores were stored in 10%-buffered formalin, dehydrated in graded series of ethanol rinses and finally embedded in glycolmethacrylate resin (Technovit-7200-VLC, Kulzer, Wehrheim, Germany).

### Synchrotron radiation microCT examination

The microCT experiments were performed at the SYRMEP beamline of ELETTRA Synchrotron Facility (Basovizza-TS, Italy) using the following settings: 1800 projections, each with 0.4 s exposure time, over a total range of 180°; pink beam with peak energy at ~19 keV; sample-detector distance at ~100 mm, resulting in (2.0 µm)^3^ isotropic voxel size in reconstructed images. Due to coherence of the synchrotron source, the intensity of recorded radiographs included phase contrast signals. The approach was based on discrimination between attenuation properties, related to the absorption index *β*, and refractive index decrement *δ* of the index of refraction *n* *=* *1 - δ + iβ* in the mineralized bone. The reconstruction was performed using Paganin’s method^49^, where the phase is retrieved by assuming that the *δ/β* ratio is constant. It was set to 100 in the present investigation.

The VG Studio MAX 1.2 (Volume Graphics, Heidelberg, Germany) software was used to generate 3D images where mass density differences within samples translate into different peaks in the gray-level scale, corresponding to the different phases (Fig. 3A). The Mixture Modeling algorithm^50^ was implemented to threshold the histograms, separating the marrow spaces from mineralized bone. For each biopsy, many disjointed and variable-sized subvolumes (with dimensions not minor than 10^6^ µm^3^) were used, as many as necessary to map the entire biopsy. Each of them was a 3D portion fully included in the sample bulk and the complete set of them allowed to achieve the complete retrieved sample mapping.

The quantitative analysis was performed with an approach based on two hierarchical levels of investigation.

In the first level, both the 3D microarchitecture and the mass density distribution of the bone samples retrieved from all patients (including controls) were studied. The microarchitectural analysis was based on structural indices usually measured for bone samples^51^ bone specific volume (BV/TV), bone specific surface (BS/BV), trabecular bone thickness (Tb.Th), trabecular bone number (Tb.Nr) and trabecular bone spacing (Tb.Sp). Furthermore, as trabecular bone varies its orientation depending on mechanical loading, we also extracted information about the presence of preferential orientation(s)^52^. The anisotropy degree index (Tb.DA), that was investigated by BoneJ Plugin^53^ of the ImageJ software^50,54,55^, varies between 0 (perfect isotropy) and 1 (strong anisotropy). Finally, the trabecular connectivity density (Tb.Conn.D) parameter was also calculated, supplying a global measure giving higher values for better-connected structures and lower values for poorly-connected ones.

Moreover, we used the reconstructed refractive index n distribution, which is linearly related to mass density, to compute the apparent bone mineral density distribution (BMDD^r^). The BMDD^r^ parameters were calculated within the mineralized domain, with the intensities normalized, for each sample, by the area under the curve. Since the reconstructed complex refractive index might be biased due to the constant ratio *δ/β*^56^, absolute values of bone mass density (calcium concentrations – Ca weight %) could not be retrieved, hereinafter the superscript *r* denoting relative values for all the parameters. However, since the different biopsies were comparable in terms of size and composition, relative difference in mass density distribution between them were appreciated. Following the Roschger approach^36^, five parameters were extracted using the PeakFit software (Systat Software, San Jose, CA): the mean relative mass density (BMDD^r^_mean_), the most frequent relative mass density value (BMDD^r^_peak_), the 0.5th (BMDD^r^_low_) and the 99.5th (BMDD^r^_high_) percentiles, and the full width at half maxima of the distribution (BMDD^r^_fwhm_).

A second level of analysis was also done, achieving information on bone ultrastructure, i.e. on morphometric properties of the osteocyte lacunae. The entire mineralized bone was indicated as total volume (TV). Each biopsy was fully mapped, investigating the mean lacunar thickness (Lc.Th), the mean lacunar volume (Lc.V), and the lacunar density (number of lacunae per total volume – Lc.Nr/TV). Furthermore, in one of the peri-dental biopsies and one of the peri-implant ones, the previous analyses were repeated at different distances of the sampling volumes from tooth/implant surface using BoneJ^53^ and Particle Analyser (https://imagej.net/Particle_Analysis) Plugins of the ImageJ software^50,54,55^.

### Histologic examination

After microCT analyses, the specimens were sectioned along their transversal direction with a high precision diamond disk at about 150 µm and ground down to about 30 µm with a Precise 1 Automated System machine (Assing, Rome, Italy)^57^. Three slices were obtained from each specimen, subsequently stained with acid fuchsin and toluidine blue before analyses.

Histological and histomorphometrical analysis of the percentages of newly formed bone, mature bone and marrow spaces was carried out using a light microscope (Laborlux S, Leitz, Wetzlar, Germany) connected to a high-resolution camera (3CCD, JVCKY-F55B, JVC, Yokohama, Japan), interfaced with a PC (Intel Pentium III 1200 MMX, Intel, Santa Clara, CA, USA) and associated with a digitizing pad (Matrix Vision GmbH, Oppenweiler, Germany) and a software package (Image-Pro Plus 4.5, Media Cybernetics Inc., Rockville, USA).

## Supplementary information

Supplementary Information.

Supplementary Information2

Supplementary Information3
