# Supplementary material for: Jawbone remodeling: a conceptual study based on Synchrotron High-resolution Tomography
Source: Sci Rep. 2020 Mar 2;10:3777. doi: 10.1038/s41598-020-60718-8 (PMC7052147; doi:10.1038/s41598-020-60718-8)
Supplement: Supplementary file 1 — Supplementary Information. [file 41598_2020_60718_MOESM1_ESM.docx]

**Jawbone remodeling: a conceptual study based on Synchrotron High-resolution Tomography**

Giovanna Iezzi^1^, Carlo Mangano^2^, Antonio Barone^3^, Federico Tirone^4^, Luigi Baggi^5,6^, Giuliana Tromba^7^, Adriano Piattelli^1,8,9^, Alessandra Giuliani^10,*^

^1^Department of Medical, Oral and Biotechnological Sciences, University of Chieti-Pescara, Chieti Scalo, CH, Italy. gio.iezzi@unich.it; apiattelli@unich.it;

^2^Private Practice, Gravedona (CO), Italy; camangan@gmail.com;

^3^Department of Medical, Surgical, Molecular and of the Critical Area Pathologies, University of Pisa, Pisa, Italy; barosurg@gmail.com;

^4^Private Practice, Cuneo, Italy; federico.tirone@gmail.com;

^5^Department of Social Dentistry, National Institute for Health, Migration and Poverty, Rome, Italy; ^6^School of Dentistry, University of Rome “Tor Vergata”, Rome, Italy; odonto.baggi@gmail.com;

^7^Elettra Sincrotrone Trieste S.C.p.A, Trieste, Italy. Giuliana.tromba@elettra.eu;

^8^Chair of Biomaterials Engineering, Catholic University of Murcia (UCAM), Murcia, Spain;

^9^Villa Serena Foundation for Research, Città Sant’Angelo (Pescara), Italy;

^10^Department of Clinical Sciences, Polytechnic University of Marche, Ancona, Italy. A.giuliani@univpm.it.

**Abstract**

Supplementary material for the article submitted to Nature Scientific Reports. This material presents movies related to microCT stack-sequences of 2D axial slices for peri-dental and peri-implant bone tissues.

**Supplemental data:**

**Movie 1**

Mv.1. Peri-dental bone. MicroCT stack-sequence of 2D axial slices (final volume of 560 × 2700 × 950 µm^3^) for a representative sample of a patient. Top: tooth root; bottom: peri-dental alveolar bone.

**Movie 2**

Mv.2. Peri-implant bone. MicroCT stack-sequence of 2D axial slices (final volume of 2500 × 1600 × 1000 µm^3^) for a representative sample of a patient. Top-left: curved interface between implant and bone after having gently removed the implant; bottom: peri-implant alveolar bone.
